# Construction of T-Cell-Related Prognostic Risk Models and Prediction of Tumor Immune Microenvironment Regulation in Pancreatic Adenocarcinoma via Integrated Analysis of Single-Cell RNA-Seq and Bulk RNA-Seq

**DOI:** 10.3390/ijms26062384

**Published:** 2025-03-07

**Authors:** Dingya Sun, Yijie Hu, Jun Peng, Shan Wang

**Affiliations:** 1 Xiangya School of Pharmaceutical Sciences, Department of Pharmacology, Central South University, Changsha 410083, China; sundingya627@163.com; 2 Department of Pharmaceutical Engineering, College of Chemistry and Chemical Engineering, Central South University, Changsha 410083, China; huyijiework@163.com

**Keywords:** scRNA-seq, bulk RNA-seq, pancreatic adenocarcinoma, T-cell, prognostic risk model, immune microenvironment

## Abstract

Pancreatic adenocarcinoma (PAAD) is a fatal malignant tumor of the digestive system, and immunotherapy has currently emerged as a key therapeutic approach for treating PAAD, with its efficacy closely linked to T-cell subsets and the tumor immune microenvironment. However, reliable predictive markers to guide clinical immunotherapy for PAAD are not available. We analyzed the single-cell RNA sequencing (scRNA-seq) data focused on PAAD from the GeneExpressionOmnibus (GEO) database. Then, the information from the Cancer Genome Atlas (TCGA) database was integrated to develop and validate a prognostic risk model derived from T-cell marker genes. Subsequently, the correlation between these risk models and the effectiveness of immunotherapy was explored. Analysis of scRNA-seq data uncovered six T-cell subtypes and 1837 T-cell differentially expressed genes (DEGs). Combining these data with the TCGA dataset, we constructed a T-cell prognostic risk model containing 16 DEGs, which can effectively predict patient survival and immunotherapy outcomes. We have found that patients in the low-risk group had better prognostic outcomes, increased immune cell infiltration, and signs of immune activation compared to those in the high-risk group. Additionally, analysis of tumor mutation burden showed higher mutation rates in patients with PAAD in the high-risk group. Risk scores with immune checkpoint gene expression and drug sensitivity analysis provide patients with multiple therapeutic targets and drug options. Our study constructed a prognostic risk model for PAAD patients based on T-cell marker genes, providing valuable insights into predicting patient prognosis and the effectiveness of immunotherapy.

## 1. Introduction

PAAD is one of the most lethal cancers due to a lack of early noticeable symptoms, leading to most patients being diagnosed at advanced stages. Current treatment options are very limited, resulting in an extremely high fatality rate [1]. Resistance to conventional therapies like chemotherapy and radiotherapy plus high recurrence rates post-surgery contribute to the bleak outcomes associated with PAAD treatment [2]. Immunotherapy, encompassing immune checkpoint inhibitors (ICIs), cellular immunotherapy, and adoptive cell therapy, has emerged as a pivotal treatment for patients with advanced PAAD [3]. Nonetheless, the considerable heterogeneity of PAAD tumors results in varying efficacy levels among patients [4], underscoring the significance of identifying reliable molecular markers and prognostic models to aid clinical treatment decisions.

Numerous clinical trials have demonstrated that the effectiveness of immunotherapy is associated with the type, quantity, and quality of T-cells present in the tumor site [5]. Unlike other tumor types, PAAD exhibits higher immunogenicity and harbors large quantities of tumor-infiltrating T-cells inside the tumor [6]. The composition and abundance of these tumor-infiltrating T-cells perform a key role in determining the effectiveness of immunotherapy and the overall prognosis of the patient. This correlation has been validated across various tumor types [7,8], underscoring the importance of investigating the status of tumor-infiltrating T-cells in PAAD for the improvement of clinical immunotherapy strategies.

**Figure 1:**
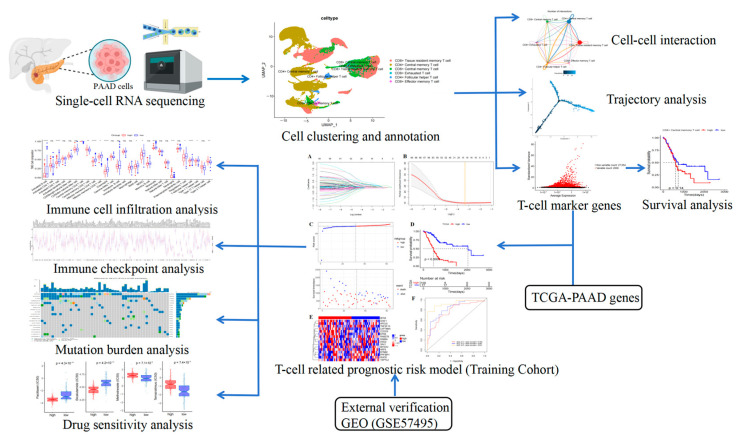
Workflow of the present study.

Considering the pivotal role of T-cells in tumor immunotherapy, comprehensively evaluating the molecular features and conditions of T-cells in individuals with PAAD could provide valuable information in forecasting the outcomes of immunotherapy [9]. In this study, the information from the PAAD dataset within the TCGA and GEO repositories was employed to establish and confirm a predictive model for PAAD patients at a cellular level. Through the amalgamation and examination of an extensive array of data from both scRNA-seq and bulk RNA sequencing (bulk RNA-seq), this study performed analysis for survival, the immune microenvironment, prognostication of response to immunotherapy, and drug susceptibility. The workflow of the whole study is shown in Figure 1. These endeavors are directed towards providing innovative perspectives within the field of immunotherapy for PAAD.

## 2. Results

### 2.1. T-Cell Subset Analysis in PAAD

**Figure 2:**
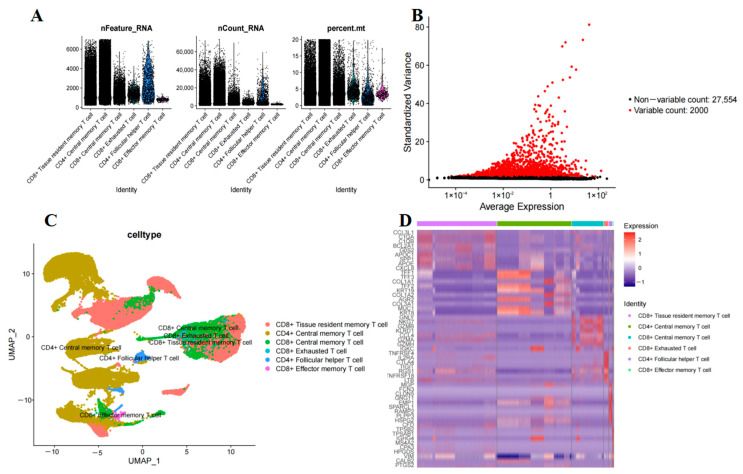
Integration and clustering of PAAD scRNA-Seq data. (**A**) Violin plot after quality control. (**B**) Volcano plot of DEGs. (**C**) T-cell type annotations. (**D**) Heat map showing top ten marker genes in 6 cell types.

A total of 8 PAAD single-cell samples (GSE197177) were analyzed in this study, capturing 59,704 cells. The single-cell dataset underwent quality control, followed by the generation of a box plot (Figure 2A) and identification of the top 2000 hypervariable genes using the ’FindVariableFeatures’ function (Figure 2B). T-cell subpopulations were manually annotated based on known marker genes from the literature [10,11], using the R package harmony to mitigate batch effects. Six T-cell subpopulations were identified: CD8^+^ tissue-resident memory T-cell (T_RM_), CD4^+^ central memory T-cell (T_CM_), CD8^+^ T_CM_, CD8^+^ exhausted T-cell (T_EX_), CD4^+^ follicular helper T-cell, and CD8^+^ effector memory T-cell (T_EF_) (Figure 2C). By employing the FindAllMarkers function for screening, a total of 2170 DEGs were identified among 1837 T-cell subpopulations (Figure 2D) (Supplementary Table S1).

### 2.2. Cell–Cell Interaction Analysis Related to T-Cells

**Figure 3:**
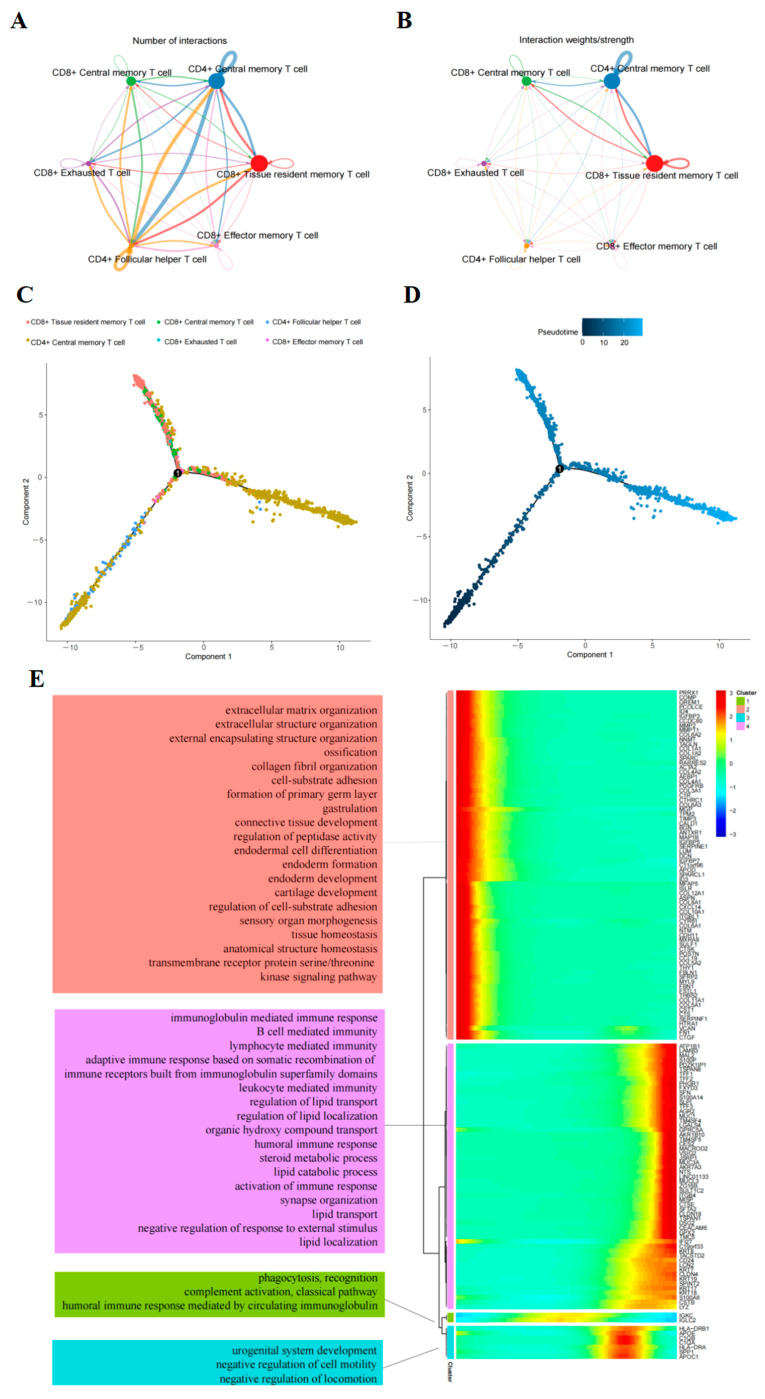
Analysis of T-cell trajectory and intercellular communication in pancreatic ductal adenocarcinoma (PAAD). (**A**,**B**) Quantification of interactions and strengths in communication networks between cells. (**C**,**D**) Diagram illustrating distinct T-cell clusters present in PAAD. (**E**) Heatmap displaying levels of dynamic gene expression over pseudo-time and the most significant Gene Ontology Biological Process term for each group.

**Figure 4:**
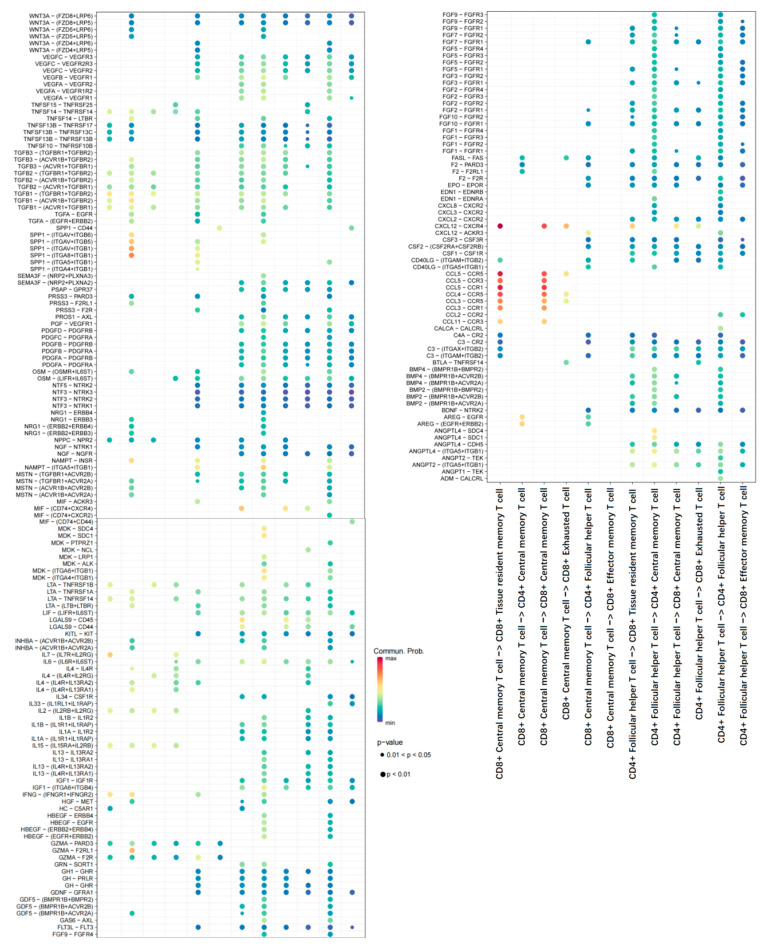
Bubble chart indicating the activity of signaling pathways across various cell populations.

Based on the annotated single-cell data, our study employed CellChaT-cell communication analysis to investigate the communication characteristics among T-cell subtypes (Figure 3A,B). Our findings revealed that CD8^+^ T_RM_ and CD4^+^ T_CM_ exhibited the highest number of interactions, particularly with the CD4^+^ follicular helper T-cell subpopulation. Notably, the strongest communication intensity was observed between CD8^+^ T_RM_ and CD4^+^ T_CM_ or CD8^+^ T_CM_. Furthermore, our analysis identified key ligand-receptor-mediated cell interactions within the CXCL12-CXCR4 and CCL5 signaling pathways (specifically the CCL5-CCR5 and CCL5-CCR1 interactions) (Figure 4).

### 2.3. Trajectory of T-Cells Maturation in PAAD

Pseudo-time analysis using Monocle to confirm T-cell differentiation status: The results revealed the initial state to be CD4^+^ T_CM_ and the final state as CD8^+^ T_RM_ (Figure 3C,D). Subsequent differentialGeneTest function analysis identified 141 significantly differential genes (DEGs) involved in the differentiation process (Figure 3E) (Supplementary Table S2). These genes, which change over developmental time, were categorized into four clusters associated with extracellular matrix organization, lymphocyte-mediated immunity, circulating immunoglobulin-mediated humoral immune response, and negative regulation of exercise, among others (Figure 3E, Supplementary Table S3).

### 2.4. Prognostic KM Curves of T-Cell Subsets

**Figure 5:**
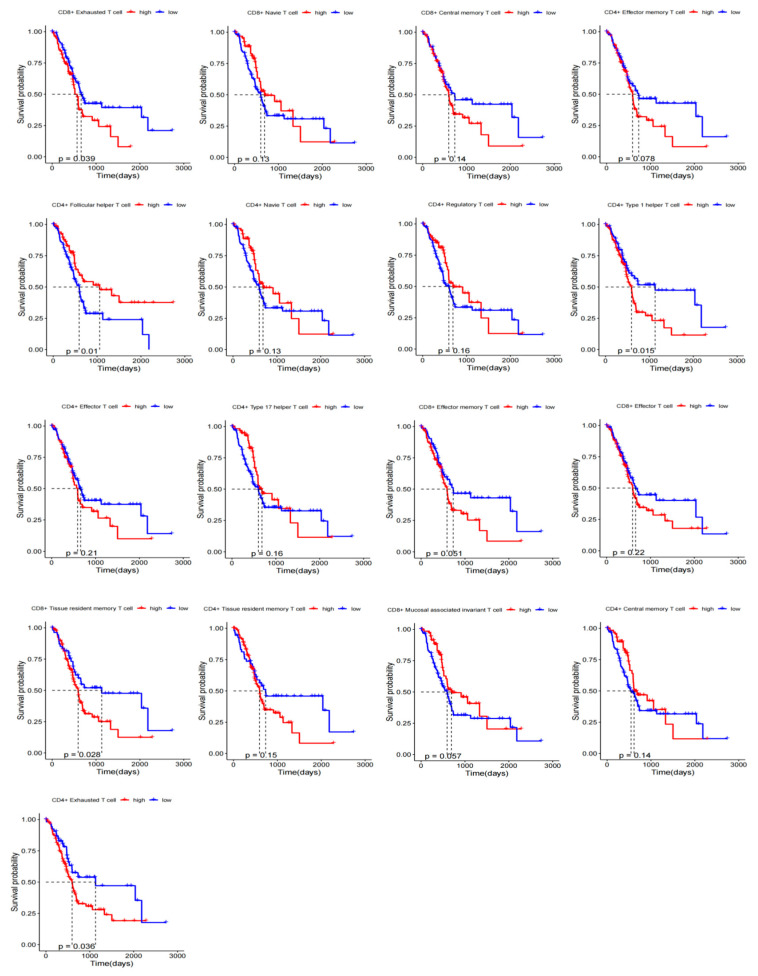
KM analysis of PAAD patients based on 17 T-cell subsets (based on best value).

To investigate the relationship between infiltration of different T-cell subtypes and OS of patients with PAAD, we analyzed 182 samples (4 normal + 178 tumors) from TCGA-PAAD data. The GSVA algorithm was utilized to estimate the infiltration levels of various T-cell subsets in PAAD samples. The surv_cutpoint function was employed to determine the optimal cutoff value, thereby categorizing each T-cell subset into high-infiltration and low-infiltration groups (Supplementary Tables S4 and S5). Subsequently, KM survival curves for 17 T-cell subtypes were generated using the OS data from TCGA-PAAD (Figure 5). Among these subtypes, CD8^+^ T_RM_, CD8^+^ T_EX_, CD4^+^ T_EX_, CD4^+^ type 1 helper T-cell, and CD4^+^ follicular helper T-cell showed significant prognostic value (*p* < 0.05).

### 2.5. Prognostic Model Construction and Validation

**Figure 6:**
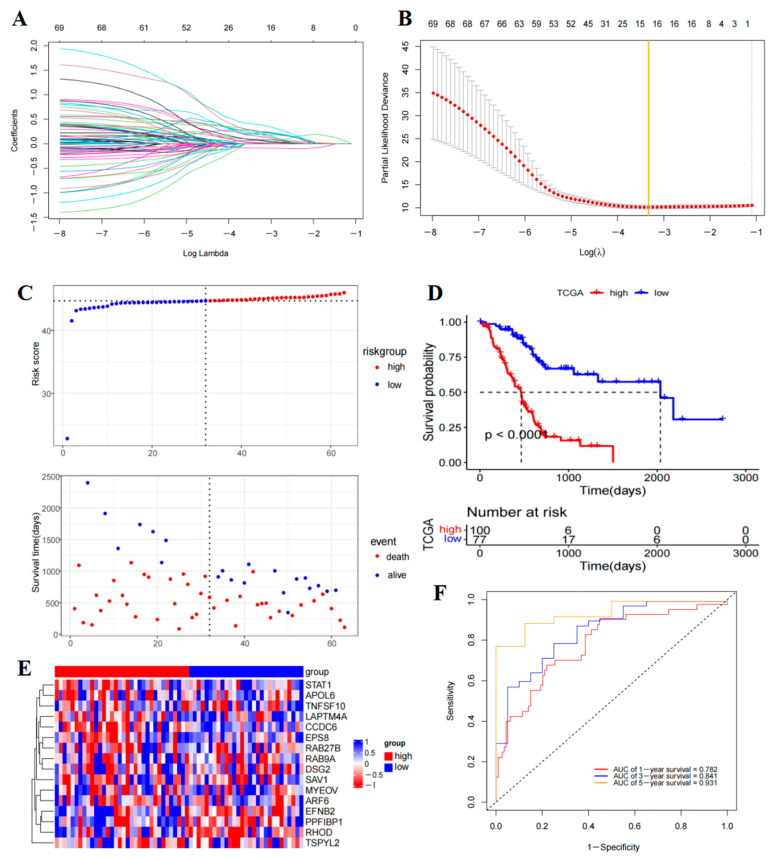
PAAD cohort was used for constructing the T-cell marker gene risk mode. (**A**,**B**) LASSO regression was performed to identify 16 DEGs with the most significant prognostic value. (**C**) Distribution of training cohort risk scores and survival status. (**D**) Survival analysis KM curves for TCGA PAAD patients based on the risk score in the training cohort. (**E**) Heatmap displaying the 16 DEGs included in the risk signature. (**F**) ROC analysis validated the predictive accuracy of the risk signature.

To investigate the survival characteristics associated with T-cell subtypes in PAAD patients, a study analyzed 1837 differentially expressed T-cell genes identified from single-cell data. Transcriptome data and OS information from the TCGA-PAAD cohort were utilized for this analysis. Specifically, expression data of 1770 T-cell marker genes and 177 PAAD tumor samples with OS information were collected. Subsequently, 69 prognostic genes were identified (*p* < 0.0001) (Figure 6A,B) (Supplementary Table S6). Using LASSO-Cox analysis, 16 genes (apolipoprotein L6 (APOL6), ADP ribosylation factor 6 (ARF6), coiled-coil domain containing 6 (CCDC6), desmosomal protein 2 (DSG2), ephrin-2 (EFNB2), epidermal growth factor receptor pathway substrate 8 (EPS8), lysosomal protein transmembrane 4A (LAPTM4A), myeloma overexpressed (MYEOV), PPFIA-binding protein 1 (PPFIBP1), ras-related protein RAB27B (RAB27B), RAS-associated protein 9a (RAB9A), RHOD, salvador homologue 1 (SAV1), signal transducer and activator of transcription 1 (STAT1), superfamily member 10 (TNFSF10), and testis-specific y-encoded-like protein 2 (TSPYL2)) were selected to construct a risk model (Supplementary Table S7). Risk scores are calculated as follows: Riskscore = RAB27B ∗ 0.082064613375332 + DSG2 ∗ 0.0104242722546218 + LAPTM4A ∗ 0.266566152963419 + CCDC6 ∗ 0.073665973664727 + PPFIBP1 ∗ 0.396298427160219 + STAT1 ∗ 0.0309412812730464 + TNFSF10 ∗ 0.261535315326191 + RAB9A ∗ 0.191697997429319 + EFNB2 ∗ 0.0173359894386158 + EPS8 ∗ 0.156990244159049 + SAV1 ∗ 0.0716166483938208 + ARF6 ∗ 0.221817418817992 + MYEOV ∗ 0.0940433794164834 + RHOD ∗ 0.204002531623839 + TSPYL2 ∗ (−0.0856756945012133) + APOL6 ∗ 0.242337988121394.

**Figure 7:**
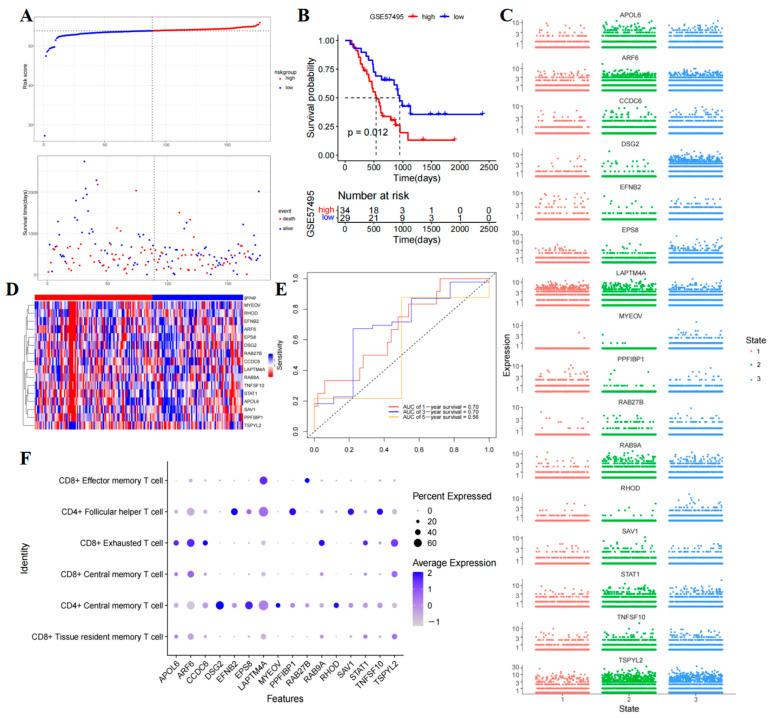
Validation of the signature risk genes associated with 16 genes. (**A**) Testing cohort distribution of risk scores and survival status. (**B**) Survival analysis KM curves in TCGA-PAAD patients based on risk score in the testing cohort. (**C**,**F**) A prognostic risk model of 16 genes associated with T-cell. (**D**) Heatmap displaying prognostic DEGs in the testing cohort. (**E**) Validation of the predictive accuracy of the risk signature through ROC analysis.

Then, TCGA-PAAD patients were categorized into high- and low-risk groups based on the risk scores (Supplementary Table S8). The KM curve indicated that patients in the high-risk group had significantly lower OS compared to those in the low-risk group (*p* < 0.0001) (Figure 6D). Additionally, visual representations such as the patient’s risk heatmap, risk score distribution map, and survival status distribution map were generated (Figure 6C–E). Subsequent analysis involved plotting ROC curves for patients, with the AUCs at one, three, and five years being 0.782, 0.841, and 0.931, respectively (Figure 6F). A comprehensive evaluation of the ROC curve demonstrated the superior predictive performance of this prognostic model. Furthermore, external validation using the GSE57495 dataset confirmed that high-risk patients had poorer OS (Figure 7A,B,D) (Supplementary Table S9), with the ROC survival results accurately predicting patient outcomes (AUCs of 0.7, 0.7, and 0.56 for one, three, and five years OS, respectively) (Figure 7E). Subsequently examining the expression of the model genes used for risk score calculation. The dot plot showed changes in the expression of 16 prognostic genes in single-cell data (Figure 7F), while the scatter plot depicted the dynamic expression of these genes across pseudo-time values (Figure 7C).

### 2.6. Correlation of the PAAD with Immune Cell Infiltration and Immune Checkpoint

Tumor-infiltrating immune cells significantly impact cancer progression and are closely associated with the clinical outcomes of patients. To better evaluate the effect of T-cell subtype-associated risk scores on the tumor microenvironment (TME), we examined immune cell infiltration in both high-risk and low-risk groups. Based on the ESTIMATE function, we assessed the immune score, stromal score, and tumor purity of the tumor samples. Our analysis indicated that there was no noteworthy disparity in the distribution of immune scores between the two risk groups (Supplementary Figure S1).

**Figure 8:**
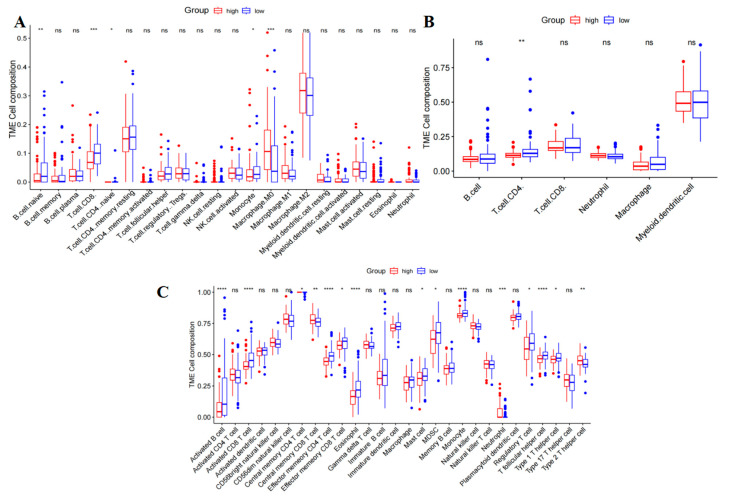
Correlation of the T-cell prognostic model genes with immune cell infiltration and immune checkpoints in the TCGA-PAAD cohort. (**A**) A comparison of the infiltration levels of 22 different immune cells between high-risk and low-risk groups was conducted. (**B**) TIMER was used to evaluate the relative abundance of six major immune cell subtypes. (**C**) The ssGSEA algorithm was employed to estimate the abundance of 24 distinct immune cell populations. (****, *p* < 0.0001; ***, *p* < 0.001; **, 0.001 < *p* < 0.01; *, 0.01 < *p* < 0.05; ns, *p* > 0.05).

We used the TIMER and CIBERSORT algorithms to study the correlation between risk scores and tumor-infiltrating immune cells. Through this analysis, we were able to quantify the levels of immune cell infiltration in our two risk groups. Additionally, we utilized the ssGSEA algorithm to evaluate the immune-related functions within these groups. Our findings revealed that low-risk patients exhibited reduced levels of activated B cells, activated CD8 T-cells, CD8 T_CM_, CD4 T_EM_, and CD8 T_EM_, eosinophils, masT-cells, myeloid cells, monocytes, Tregs cells, and type 1 helper T-cells (Figure 8C). Additionally, CIBERSORT and TIMER analyses were conducted to explore differences in immune cell levels between the two risk groups (Figure 8A,B). The results indicated that the levels of most immune memory cells (including Naive B Cell, CD4 T-cell, CD8 T-cell, Naive CD4 T-cell, and Monocyte) were significantly elevated in the low-risk group, whereas M0 macrophages were more highly expressed in the high-risk group. Collectively, these findings indicate the presence of an immune activation state in the low-risk group, possibly accounting for its more favorable prognostic outcome.

**Figure 9:**
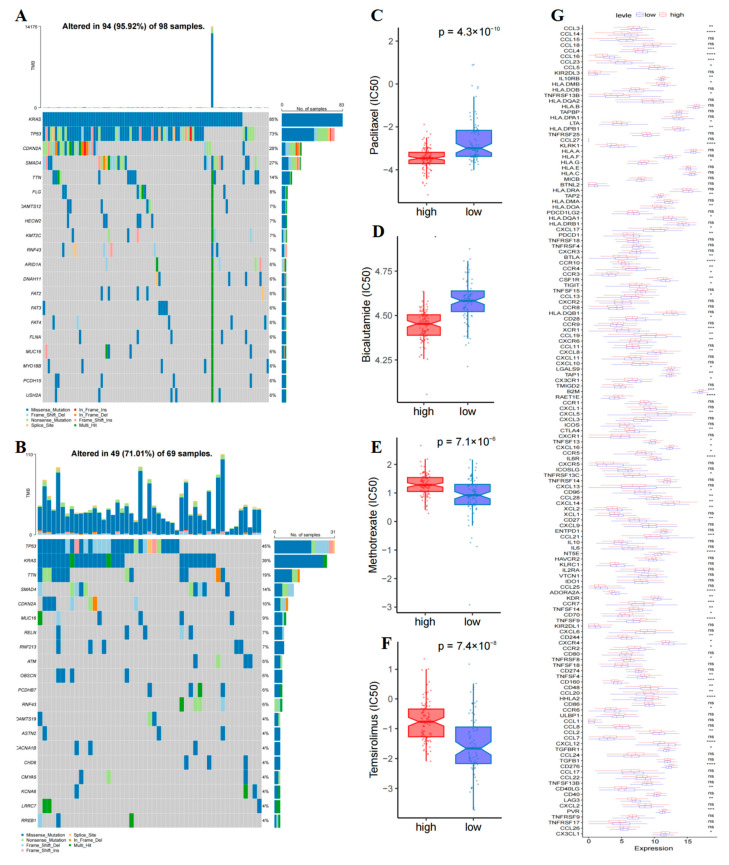
Analysis of T-cell prognostic model genes’ correlation with immune checkpoints in the TCGA cohort and assessment of tumor mutations and IC50 in the two risk categories. (**A**,**B**) Graph illustrating risk scores and TMB. (**C**–**F**) Box plots of IC50 predicted values for four drugs. (**G**) Evaluation of immune checkpoint levels in the two risk groups. (****, *p* < 0.0001; ***, *p* < 0.001; **, 0.001 < *p* < 0.01; *, 0.01 < *p* < 0.05; ns, *p* > 0.05).

Our study focused on examining how risk scores are related to immune checkpoint expression levels when it comes to immune checkpoint inhibitor (ICI) blockade therapy. The results showed that patients with low-risk scores had higher levels of immune checkpoint expression in comparison to those with high-risk scores, suggesting that individuals categorized in the low-risk category could potentially have a heightened response to ICI therapy (Figure 9G).

### 2.7. Analyses of Tumor Mutation and Half-Inhibitory Centration

To further elucidate the mechanism of tumor development in patients with PAAD, we analyzed the tumor mutation burden (TMB) for both low- and high-risk groups using TCGA somatic mutation data. KRAS, TP53, CDKN2A, SMAD4, and TTN were identified as genes with the highest mutation rates in the high-risk group (Figure 9A), while TP53, KRAS, TTN, SMAD4, and CDKN2A were found to have the highest mutation rates in the low-risk group (Figure 9B). High-risk group patients showed a higher TMB in comparison to the low-risk group. We utilized the TIDE algorithm to predict ICI responsiveness based on risk scores, and the findings revealed no significant difference in the TIDE score distribution between the two groups (Supplementary Figure S2). Furthermore, we assessed the variation in sensitivity to chemotherapy drugs between the high- and low-risk groups using the pRRophetic R package. Our analysis identified significant differences in IC50 concentrations of 100 out of 138 anti-tumor drugs between the two groups (Table 1). Additionally, we present the IC50 value distribution box plot of four drugs in Figure 9C–F, with detailed drug sensitivity analysis results available in the Supplementary File (Supplementary Table S10). Notably, the results indicated that the commonly used chemotherapeutic drugs paclitaxel, doxorubicin, and sorafenib were sensitive to patients in the low-risk group. While in the high-risk group, drugs like methotrexate, cytarabine, and mitomycin were sensitive to patients.

## 3. Discussion

Patients with PAAD often face a grim prognosis compared to other cancer types, highlighting the importance of effective treatment options. Immunotherapy has emerged as a crucial approach for managing advanced PAAD [3], with T-cells playing a pivotal role in determining treatment outcomes. The TME also significantly impacts cancer progression and the efficacy of immunotherapy. Therefore, understanding the complications of TME regulation in PAAD is significant for developing accurate prognostic models.

This study seeks to explore the significance of different T-cell subsets and their associated genes in predicting patient outcomes and responses to immunotherapy in PAAD. Heterogeneity analysis of PAAD was conducted using the GSE197177 scRNA-seq dataset. Through annotation based on T-cell subtype marker genes, a total of six T-cell subpopulations were identified: CD8^+^ T_RM_, CD4^+^ T_CM_, CD8^+^ T_CM_, CD8^+^ T_EX_, CD4^+^ follicular helper T-cell, and CD8^+^ T_EF_ cell. Intercellular communication analysis revealed that both CD8^+^ T_RM_ and CD4^+^ T_CM_ showed more frequent communication with the CD4^+^ follicular helper T-cell subpopulation. Notably, the strongest total communication intensity was observed between CD8^+^ T_RM_, CD4^+^ T_CM_, and CD8^+^ T_CM_, with intercellular communication mediated through the CXCL12-CXCR4 and CCL5 signaling pathways. It is important to highlight that the CXCR4 pathway, a commonly overexpressed chemokine receptor in various human malignancies, regulates T-cell differentiation within tumors, determining the retained lymphocyte pool, and is highly expressed in late memory subpopulations [12]. Recent studies have shown that inhibiting CXCR4 or CXCL12 can enhance tumor immunity by promoting CD8^+^ T-cell retention [13]. The analysis of T-cell trajectory reveals that 141 significantly different genes are involved in the differentiation process of T-cell subsets, particularly related to an extracellular matrix organization, lymphocyte-mediated immunity, circulating immunoglobulin-mediated humoral immune response, and negative regulation of motility, and is involved in the regulation of differentiation of CD8^+^ T_RM_ and CD4^+^ T_CM_. Memory T-cells (T_M_) are crucial in cell-mediated immune responses, persisting long after antigen elimination. Upon re-exposure to the antigen, they rapidly proliferate, exert effector functions, and release cytokines [14]. Non-circulating CD8^+^ T_RM_ offers optimal immunity against infection and has been shown to play a role in immunosurveillance in cancer [15]. For instance, CD8^+^ T_RM_ cells in various solid tumor types like lung and breast cancer (BC) can directly kill tumor cells, control tumor growth, and impact patient prognosis and treatment strategies [7,8]. The transformation of CD4^+^ T_CM_ cells into CD8^+^ T_RM_ cells in PAAD tissue appears to be a favorable occurrence, suggesting potential benefits in future PAAD immunotherapy by harnessing the specific anti-tumor effects of these T_M_.

We analyzed infiltration levels of different T-cell subsets in tissue samples from patients with PAAD to assess their impact on patient survival rates. Data from 182 samples (4 adjacent cancer tissue samples and 178 cancer tissue samples) sourced from TCGA-PAAD was utilized. Results from KM curve analysis revealed a significant association between infiltration levels of CD8^+^ T_RM_, CD8^+^ T_EX_, CD4^+^ T_EX_, CD4^+^ type one helper T-cells, and CD4^+^ follicular helper T-cells with the prognosis of PAAD patients. Specifically, higher infiltration of CD4^+^/CD8^+^ T_EX_ was linked to lower OS rates, whereas lower levels of CD8^+^ T_RM_, CD4^+^ follicular helper T-cells, and CD4^+^ type one helper T-cells were associated with reduced survival rates. Given the crucial role of these T-cell subsets in tumor response, further research is needed to fully understand their impact on cancer treatment [14], particularly in the context of immunotherapy.

We created a prognostic risk model utilizing data from the METABRIC cohort to explore the relationship between T-cells and clinical outcomes in patients with PAAD. This model relies on genes that serve as markers for T-cells, which were verified using the GEO cohort and displayed excellent predictive capability. The AUC values for the training group were impressive, with scores of 0.782 at one year, 0.841 at three years, and 0.931 at five years. Further validation using the GSE57495 dataset affirmed the prognostic model’s reliability and consistency over time. Additionally, sixteen independent prognostic genes including APOL6, ARF6, CCDC6, DSG2, EFNB2, EPS8, LAPTM4A, MYEOV, PPFIBP1, RAB27B, RAB9A, RHOD, SAV1, STAT1, TNFSF10, and TSPYL2 were identified. Notably, APOL6, a known tumor suppressor gene, emerged as a potential prognostic biomarker for various cancers. KM analysis of clinical data revealed that high expression of APOL6 was associated with improved survival rates in patients with pancreatic, ovarian, lung, liver, and gastric cancers who underwent immunotherapy [16]. ARF6 plays a crucial role in regulating vesicle transport and membrane lipid remodeling, its overexpression in cancers like pancreatic and BC contributes to tumor invasion, metastasis, and immune evasion [17]. Research indicates that ARF6 can influence gemcitabine resistance in PAAD through various pathways [18], impacting patient survival rates [19]. Additionally, CCDC6, a substrate of ataxia telangiectasia mutated protein (ATM), is involved in apoptosis, DNA damage, and repair responses. Mutations and gene rearrangements in CCDC6 have been observed in different cancer types, leading to resistance against radiation and chemotherapy [20]. Another key player, DSG2, a transmembrane protein, modulates tumor invasion and metastasis by regulating intercellular adhesion, thus, affecting tumor malignancy and prognosis [21]. Studies suggest that the decreased DSG2 levels in PAAD may influence its invasion and metastasis [22]. Lastly, EFNB2 is highly expressed in certain tumor types and is linked to disease progression. Knockdown of EFNB2 in PAAD cells has been shown to reduce cell migration and invasion by inhibiting epithelial-to-mesenchymal transition [23]. EPS8 is a scaffolding protein that regulates proliferation and receptor trafficking in solid tumors like PAAD, and its high expression mediates tumor cell migration and disease progression [24]. In BC, EPS8 promotes cell growth, migration, and invasion through pathways involving extracellular signaling-regulated kinase (ERK), matrix metalloproteinase-9 (MMP9), P53, and EMT-like transformation [25]. LAPTM4A is highly expressed in a variety of tumor tissues and is considered a significant risk factor for patients with PAAD [26]. LAPTM4A’s relationship with immune cell infiltration and tumor drug sensitivity has implications for patient prognosis [27]. MYEOV is identified as an oncogene that exhibits abnormal overexpression across various cancer types, particularly linked to the diminished survival rates of PAAD patients. It serves as a pivotal target for PAAD therapy [28]. Previous studies have substantiated that MYEOV stimulates the proliferation of PAAD cells by augmenting the activation of multiple oncogenic pathways [29]. Conversely, the deletion of MYEOV hampers the metastatic and proliferative capacities of PAAD cells. Moreover, the heightened expression of PPFIBP1 is strongly associated with glioblastoma invasion and unfavorable patient outcomes, positioning it as a potential target for glioma therapy [30]. RAB27B regulates exosome secretion in a variety of tumors, including PAAD, hepatocellular carcinoma, and colorectal cancer (CRC), and its high expression correlates with tumor cell proliferation and invasion and poor prognosis. Notably, the downregulation of Rab27B can significantly impede the invasion and proliferation of PANC-1 cells [31]. Additionally, the knockdown of RAB9A diminishes the proliferation and invasion of melanoma cells, while also fostering apoptosis [32]. RHOD plays a crucial role in the tumor invasion process by regulating fiber formation, cell movement, and other essential processes [33]. The tumor suppressor SAV1 is downregulated in various tumor types, leading to tumor invasion, disease progression, and ultimately poor prognosis. Research indicates that SAV1 can impede CRC progression by inhibiting the YAP-Akt-mTOR signaling pathway [34], whereas reduced SAV1 expression can enhance PAAD invasion and migration [35]. Furthermore, STAT1 is generally recognized as a tumor suppressor. Studies have found that high STAT1 expression is associated with unfavorable OS rates in patients with PAAD, renal cancer, lung adenocarcinoma, and other malignancies. Conversely, elevated STAT1 levels benefited long-term survival in patients with ovarian cancer, sarcoma, and melanoma [36]. TNFSF10 plays a vital role in regulating the antiviral immune response in BC [37]. TSPYL2 plays an inhibitory role in a variety of cancers, and its reduced expression is associated with multiple lymph node metastases in thyroid cancer tissues [38]. Moreover, TSPYL2 may inhibit thyroid cancer progression by modulating SIRT1/AKT signaling. Studies have indicated that TSPYL2 can reduce gefitinib (GEF) resistance in CRC by suppressing sirt1-mediated FOXO3 deacetylation, making it a promising target for anti-tumor therapy [39]. Furthermore, an analysis of the expression changes of key genes used for constructing risk score characteristics during cell differentiation revealed that most model genes exhibited an increasing trend in expression. In summary, APOL6, ARF6, CCDC6, DSG2, EFNB2, EPS8, LAPTM4A, MYEOV, PPFIBP1, RAB27B, RAB9A, RHOD, SAV1, STAT1, TNFSF10, and TSPYL2 are noteworthy as prognostic indicators of PAAD.

The study involved an examination of immune cell levels in two distinct risk groups, utilizing computational tools CIBERSORT and TIMER. The results of the investigation indicated that individuals in the low-risk group showed elevated expression levels of key immune memory cells, including the Naive B cell, CD4 T-cell, CD8 T-cell, Naive CD4 T-cell, and Monocyte. In contrast, the high-risk group exhibited higher expression of M0 macrophages, which are associated with a more pro-inflammatory response. Research indicates that M0 macrophages can polarize into M1 and M2 states in response to microenvironmental signals, with a tendency towards the M2 phenotype in typical tumor microenvironments characterized by hypoxia, high lactate, inflammation, and oxidative stress [40]. This polarization promotes immunosuppression and tumor progression, with studies confirming that the presence of M2 macrophages in the PAAD microenvironment accelerates tumor progression and leads to poor prognosis [41,42,43]. It was observed that the low-risk group demonstrated increased levels of immune memory cells, suggesting a potentially more robust immune response and better overall immune system function. These findings provide valuable insights into potential differences in immune cell profiles between individuals at varying levels of risk, highlighting the importance of understanding the role of specific immune cell populations in disease susceptibility and progression. The comprehensive analysis of risk score characteristics and immune checkpoint expression has identified a total of 73 genes that differ significantly between the two risk groups. This substantial discovery suggests a strong correlation with the outcomes of immunotherapy. Nonetheless, further investigation is needed to understand the relationship between immune checkpoint molecule expression and the success of immunotherapy in PAAD patients comprehensively. By leveraging data on somatic mutations from TCGA, we analyzed the mutation gene profiles across the two risk groups. This analysis identified a higher frequency of gene mutations in the high-risk group, notably involving pivotal genes such as KRAS, TP53, CDKN2A, SMAD4, and TTN, which are crucial drivers in the progression of PAAD [44,45,46]. We utilized the TIDE algorithm to anticipate how risk scores would respond to ICIs. The results of the analysis did not show any notable disparities in the distribution of TIDE scores between the two groups, suggesting that the effectiveness of these genes in forecasting the response to ICIs in PAAD patients remains uncertain. Drug resistance in PAAD patients poses a significant treatment challenge [2], and forecasting drug sensitivity using the GDSC database allows for personalized treatment options that consider individual differences in response to chemotherapeutic agents. As a result, our findings offer multiple drug treatment options for PAAD patients, although the ultimate efficacy requires validation through clinical trials.

Our study has several limitations. The data analyzed in this paper are derived entirely from public datasets. Therefore, extensive preclinical research and validation in prospective clinical trials are necessary to confirm our risk model and conclusions. Additionally, due to the diverse sources of the PAAD data cohort that we examined, variability within tumors or among patients is expected. Therefore, extensive prospective clinical studies are required to confirm its accuracy. Notwithstanding these limitations, our study could potentially offer valuable insights for clinical decision-making in PAAD patients undergoing immunotherapy.

## 4. Materials and Methods

### 4.1. Data Source and Preprocessing

The TCGA-PAAD data (https://www.cancer.gov/ccg/research/genome-sequencing/tcga, accessed on 14 May 2024), including mutation data, clinical information, and overall survival time, was downloaded from the TCGA official website using the R TCGAbiolinks package (https://bioconductor.org/packages/release/bioc/html/TCGAbiolinks.html, accessed on 14 May 2024) [47]. A total of 182 samples were included in the analysis, with one sample labeled as ‘-11A’ considered normal tissue and the remaining samples as cancer tissue. Among these samples, 177 cancer samples with overall survival (OS) prognostic information were used for model construction and analysis. Eight samples from the GEO database [48] (http://www.ncbi.nlm.nih.gov/geo/, accessed on 15 May 2024) were downloaded from the PAAD scRNA-seq dataset GSE197177 for T-cell subset identification. Additionally, the GSE57495 dataset was obtained as a validation set, from which cancer samples with prognostic information were extracted. Among these, 63 tumor samples were selected for further model verification. We directly downloaded the processed and normalized probe expression matrix and obtained the corresponding platform annotation file to facilitate the conversion of probes into gene symbols. For cases where different probes correspond to the same gene symbol, we calculated the average value to represent the expression value of the gene for subsequent analysis.

### 4.2. T-Cell Subtypes and Differential Gene Expression Analysis

Cellular quality control and cluster analysis of scRNA-seq data were conducted utilizing the R package Seurat [49] (https://github.com/satijalab/seurat, accessed on 17 May 2024). Cells that had fewer than 200 genes, more than 7000 genes in total, and over 20% mitochondrial genes were excluded. The top 2000 hypervariable-expressing genes were identified using the FindVariableFeatures function. The R package harmony was employed for sample integration to mitigate batch effects. Subsequently, cell clusters were manually annotated based on established T-cell subtype marker genes [10,11], and differentially expressed genes (DEGs) within their subpopulations were identified.

### 4.3. Cell–Cell Communication Analysis

The CellChat package [50] (https://github.com/sqjin/CellChat, accessed on 17 May 2024) was utilized to analyze various connection patterns among different T-cell types, enabling the construction of cellular communication networks and interaction relationships within T-cell subpopulations.

### 4.4. Pseudo-Time Trajectories Analysis

The pseudo-chronological analysis was conducted using the monocle package [51] (https://bioconductor.org/packages/release/bioc/html/monocle.html, accessed on 17 May 2024) to forecast the differentiation relationships among subpopulations within the T-cell population. This analysis helped infer cell trajectories and pseudo-time, as well as identify potential genes involved in the differentiation process.

### 4.5. Prognostic KM Curves of T-Cell Subsets

Utilizing TCGA-PAAD data, standardized data was obtained using the DESEq2 package [52] (https://bioconductor.org/packages//2.12/bioc/html/DESeq2.html, accessed on 17 May 2024) for subsequent analysis. The GSVA algorithm [53] was used to estimate the infiltration content of T-cell subpopulations, and these subpopulations were divided into high- and low-infiltration groups based on the optimal cutoff value. Survival analysis was performed with the survival package [54] (https://cran.r-project.org/web/packages/survival/index.html, accessed on 17 May 2024), and OS rates were compared between the high and low infiltration groups using the Kaplan–Meier (KM) method [55]. The aim was to assess the relationship between the infiltration content of T-cell subpopulations and the prognosis of OS rates.

### 4.6. Prognostic Model Construction and Validation

The glmnet package (https://cran.r-project.org/web/packages/glmnet/index.html, accessed on 18 May 2024) was utilized to conduct LASSO Cox regression analysis on the DEGs identified in single-cell T-cell subpopulations. This analysis aimed to identify the most relevant feature genes and develop a prognostic risk model. The risk score for each tumor sample in the TCGA-The PAAD dataset was calculated using the following formula ($\mathrm{Risk}\;\mathrm{Score}=\sum_{i=0}^{k}\beta i*\exp i^{1}$, “expi” refers to the expression level of a specific gene, and “βi” refers to its corresponding coefficient). Following categorization, the samples were classified into high-risk and low-risk categories according to the optimal risk score values. Survival prognostic curves were then generated using the survival R package to examine the correlation between the designated risk groups and actual survival outcomes. To assess statistical significance, the log-rank test was employed in the analysis for the survival prognostics. Furthermore, the prognostic value of the centralized risk model was assessed through the area under the curve (AUC) and receiver operating characteristic (ROC) curve. To ensure the accuracy and reliability of the risk model, external validation was conducted using the GSE57495 cohort.

### 4.7. Immune Cell Infiltration Analysis

The ESTIMATE R package [56] (https://r-forge.r-project.org/projects/estimate/, accessed on 19 May 2024) was utilized to calculate the sample matrix score, immune score, and tumor purity. Additionally, the immune infiltration and tumor microenvironment (TME) was evaluated using CIBERSORT [57], TIMER [58], and ssGSEA [59] algorithms. The association between risk score and immune checkpoint expression was investigated through the Tumor and Immune System Interaction Database (TISIDB) (http://cis.hku.hk/TISIDB, accessed on 22 May 2024).

### 4.8. Analysis of Drug Sensitivity and Immunotherapy Sensitivity

Data from TCGA was examined to assess the mutation rates of genes in high-risk versus low-risk cohorts. TIDE [60] (http://tide.dfci.harvard.edu/, accessed on 26 May 2024) algorithms were then applied to forecast how risk scores would react to Immune Checkpoint Inhibitors (ICIs). Moreover, the Genomics of Drug Sensitivity in Cancer (GDSC) (https://www.cancerrxgene.org/, accessed on 28 May 2024) repository was utilized to estimate the susceptibility of individual specimens to specific chemotherapy medications, and utilize the pRRhetic package to compare the half-maximal inhibitory concentration (IC50) of these drugs [61] (https://github.com/paulgeeleher/pRRophetic, accessed on 28 May 2024).

### 4.9. Statistical Analysis

Survival differences among various risk groups were assessed using KM survival analysis. Predictive models were developed through LASSO regression and Cox regression analyses. To examine the relationship with continuous variables across the two groups, the Wilcoxon rank-sum test was implemented. Statistical analysis was conducted using R software version 4.2.2, with a significance level set at *p* < 0.05. Additionally, significance levels were defined as follows: *p* < 0.05 (*), *p* < 0.01 (**), and *p* < 0.001 (***). A *p*-value < 0.05 was chosen for analyzing drug sensitivity.

## 5. Conclusions

This study utilized both scRNA-seq and bulk RNA-seq to investigate the T-cell profile in PAAD. Using risk scores derived from the sequencing data, the study was able to predict various aspects such as prognosis, immune microenvironment, tumor mutations, and drug sensitivity in PAAD patients. These findings offer potential new targets for treatment and improve clinical management strategies for PAAD patients. The results of this study require further experimentation and clinical practice for validation.

## Figures and Tables

**Table 1:** Comparison of the drug sensitivity between low-risk group and high-risk group.

	Low-Risk Group	High-Risk Group
A.443654	Better	
A.770041	Better	
AKT.inhibitor.VIII	Better	
AZD.0530	Better	
BI.2536	Better	
BIBW2992	Better	
Bicalutamide	Better	
BMS.509744	Better	
BMS.536924	Better	
Bortezomib	Better	
Bryostatin.1	Better	
CGP.60474	Better	
CGP.082996	Better	
CHIR.99021	Better	
CI.1040	Better	
CMK	Better	
Dasatinib	Better	
Epothilone.B	Better	
Erlotinib	Better	
FH535	Better	
FTI.277	Better	
GNF.2	Better	
GSK.650394	Better	
JW.7.52.1	Better	
Lapatinib	Better	
LFM.A13	Better	
NSC.87877	Better	
NVP.TAE684	Better	
Paclitaxel	Better	
PD.0325901	Better	
PF.562271	Better	
PHA.665752	Better	
RDEA119	Better	
Thapsigargin	Better	
VX.680	Better	
WH.4.023	Better	
WZ.1.84	Better	
X17.AAG	Better	
Z.LLNle.CHO	Better	
AUY922	Better	
AZ628	Better	
AZD6244	Better	
Bexarotene	Better	
Bleomycin	Better	
BMS.754807	Better	
Docetaxel	Better	
GW843682X	Better	
KIN001.135	Better	
MG.132	Better	
Midostaurin	Better	
Rapamycin	Better	
S.Trityl.L.cysteine	Better	
Sorafenib	Better	
XMD8.85	Better	
AP.24534		Better
BI.D1870		Better
BX.795		Better
Cytarabine		Better
DMOG		Better
Elesclomol		Better
GDC.0449		Better
GDC0941		Better
JNK.9L		Better
Mitomycin.C		Better
ABT.263		Better
ABT.888		Better
AG.014699		Better
AMG.706		Better
ATRA		Better
Axitinib		Better
AZD.2281		Better
AZD8055		Better
BIRB.0796		Better
Camptothecin		Better
CCT007093		Better
CEP.701		Better
EHT.1864		Better
IPA.3		Better
JNK.Inhibitor.VIII		Better
Lenalidomide		Better
Metformin		Better
Methotrexate		Better
MK.2206		Better
Nilotinib		Better
NU.7441		Better
Nutlin.3a		Better
NVP.BEZ235		Better
PD.173074		Better
PD.0332991		Better
PLX4720		Better
QS11		Better
Salubrinal		Better
SB590885		Better
SL.0101.1		Better
Temsirolimus		Better
TW.37		Better
Vorinostat		Better
VX.702		Better
X681640		Better
ZM.447439		Better

## Data Availability

The datasets presented in this study can be found in online repositories. The names of the repository/repositories and accession number(s) can be found in the article/Supplementary Materials.

## Supplementary Materials

The following supporting information can be downloaded at: https://www.mdpi.com/article/10.3390/ijms26062384/s1.
