# Supplementary figures and images for: Construction of T-Cell-Related Prognostic Risk Models and Prediction of Tumor Immune Microenvironment Regulation in Pancreatic Adenocarcinoma via Integrated Analysis of Single-Cell RNA-Seq and Bulk RNA-Seq
Source: Int J Mol Sci. 2025 Mar 7;26(6):2384. doi: 10.3390/ijms26062384 (PMC11942068; doi:10.3390/ijms26062384)

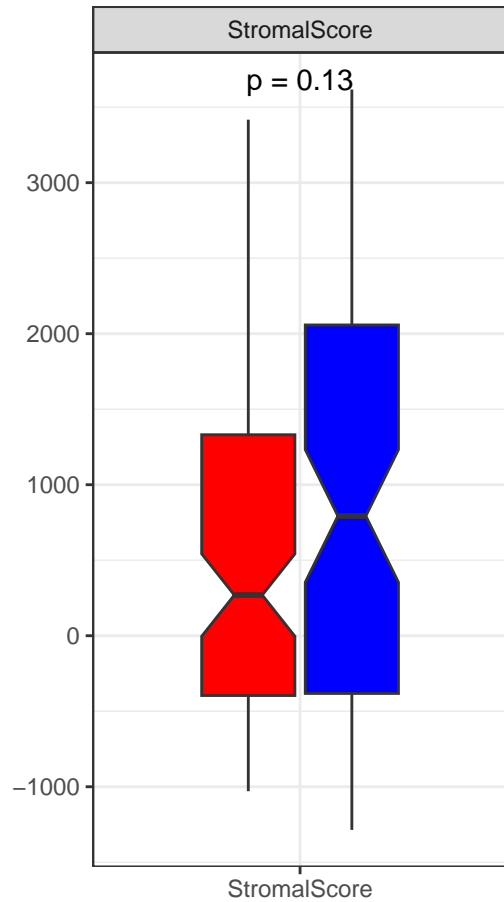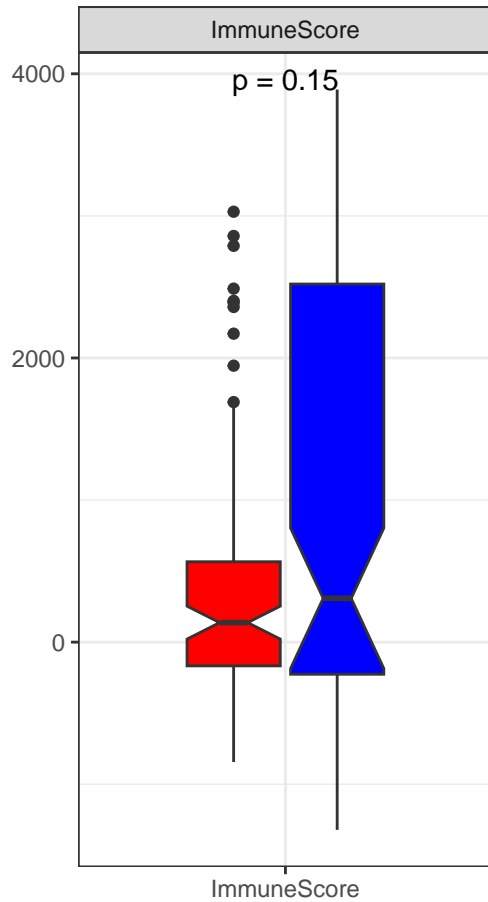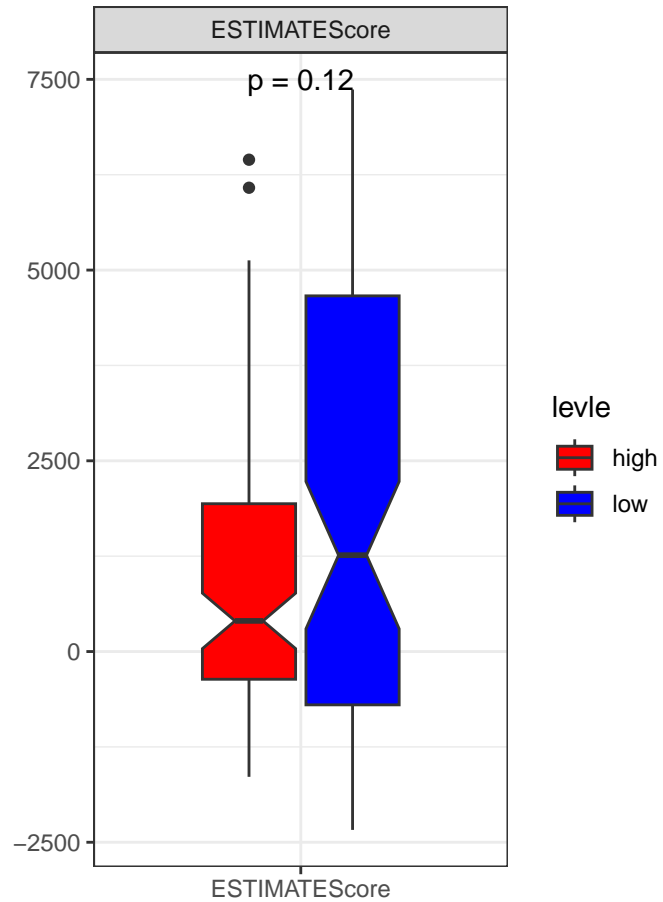

Supplement: Supplementary file 1 [file ijms-26-02384-s001.zip › Supplementary Figure S1/ESTIMATE_boxplot.pdf]

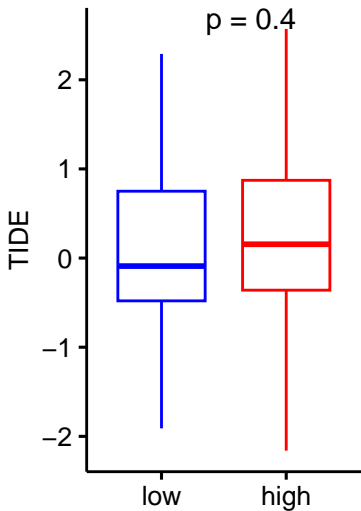

Supplement: Supplementary file 1 [file ijms-26-02384-s001.zip › Supplementary Figure S2/TIDE.pdf]
